# Personal

**Published:** 1918-12

**Authors:** 


### PERSONAL

Dr. Harry O. Maldinger of N. Tonawanda, in service in France, writes of the subsidence of influenza among troops and of his own recovery after an attack.

Dr. Bert John Bixby of Buffalo has been commissioned captain in the Medical Corps of the Army.

Dr. Wm. Stephen Driscoll of Buffalo has been commissioned captain, M. C.

Dr. Franklin C. Gram, Acting Health Commissioner of Buffalo, was forced to leave the work of the dept. in temporary charge of Dr. George S. Staniland for a few days during the influenza epidemic, on account of tachycardia due to overwork.

Capt. Ulysses B. Stein of Buffalo was ordered to Camp Greenleaf for instruction, late in Oct.

Capt. Henry R. Lohnes of Buffalo was ordered to duty at General Hospital No. 22 at Richmond, late in Oct.

Dr. George E. Blackham of Dunkirk sustained a fracture of the left shoulder in an automobile collision Oct. 8 and was treated at the Brooks Memorial Hospital.

Col. Albert H. Briggs of Buffalo has moved his office to 1312 Main St. and his residence to the Buckingham, Allen and Mariner Sts.

Dr. Chaffee of Brooklyn has moved to Binghamton and opened an office for the practice of surgery at 100 Hawlet St. He has been on the staff of the Polyclinic of N. Y. City for 25 years and was the founder and a former president of the N. Y. and N. E. Assn. of Railway Surgeons.

Dr. Anna P. Walsh of Buffalo, has completed her interneship at Bellevue and returned to Buffalo.

Dr. W. W. Plummer of Buffalo who went to France a year ago, has been promoted to the rank of major and assigned as consultant to a group of base hospitals in the St. Mihiel sector.

